# Exploring the Potential of Recycled Polymers for 3D Printing Applications: A Review

**DOI:** 10.3390/ma17122915

**Published:** 2024-06-14

**Authors:** Rachel Djonyabe Habiba, Cândida Malça, Ricardo Branco

**Affiliations:** 1 Centre for Rapid and Sustainable Product Development (CDRSP), Polytechnic Institute of Leiria (IPL), 2430 Marinha Grande, Portugal; rachel.d.habiba@ipleiria.pt (R.D.H.); candida@isec.pt (C.M.); 2 Coimbra Institute of Engineering (ISEC), Polytechnic Institute of Coimbra (IPC), Rua Pedro Nunes–Quinta da Nora, 3030-199 Coimbra, Portugal; 3 CEMMPRE, ARISE, Department of Mechanical Engineering, University of Coimbra, Rua Luis Reis Santos, 3030-788 Coimbra, Portugal

**Keywords:** recycled materials, recycled polymers, 3D printing, sustainability, additive manufacturing, circular economy

## Abstract

The integration of recycled polymers into additive manufacturing (AM) processes offers a promising opportunity for advancing sustainability within the manufacturing industry. This review paper summarizes existing research and developments related to the use of recycled materials in AM, focusing on distinct polymers, such as polylactic acid (PLA), polyethylene terephthalate (PET), and acrylonitrile butadiene styrene (ABS), among others. Key topics explored include the availability of recycled filaments on the market, challenges associated with material variability and traceability, and efforts toward establishing ethical product standards and sustainability characterization methodologies. Regulatory considerations and standards development by organizations such as ASTM and ISO are discussed, along with recommendations for future advancements in improving the sustainability of filament recycling and achieving net-zero emissions in AM processes. The collective efforts outlined in this paper underscore the potential of recycled polymers in AM to foster a more sustainable and environmentally friendly manufacturing industry.

## 1. Introduction

Three-dimensional (3D) printing, also known as additive manufacturing (AM), is a process of deposing material governed by the International Organization for Standardization (ISO) and American Society for Testing and Materials (ASTM) standards [1] and is being increasingly used. Three-dimensional printers are easy to use and faster to learn compared to computer numerical control (CNC) or other conventional manufacturing machines. They show numerous advantages over conventional manufacturing for technological advancements, considering the environmental impact, since they use just the amount of material needed for production, leading to a reduction in material waste, unlike traditional manufacturing which generates more material waste. To address environmental issues, there is a growing interest in developing sustainable solutions for 3D printing. This includes, among others, the use of recycled materials, the development of biobased materials, and the optimization of printing parameters.

The recycling of materials in the additive manufacturing industry has a significant impact on environmental preservation by reducing the need for new raw materials [2]. Recycled components actively contribute to the conservation of natural resources, helping to face resource reduction. Moreover, the production of most recycled materials requires less energy than their virgin counterparts used in conventional methods, leading to a more sustainable approach to energy consumption in 3D printing processes. Additionally, materials recycled from landfills help in waste reduction and pollution mitigation, promoting a circular economy.

**Figure 1:**
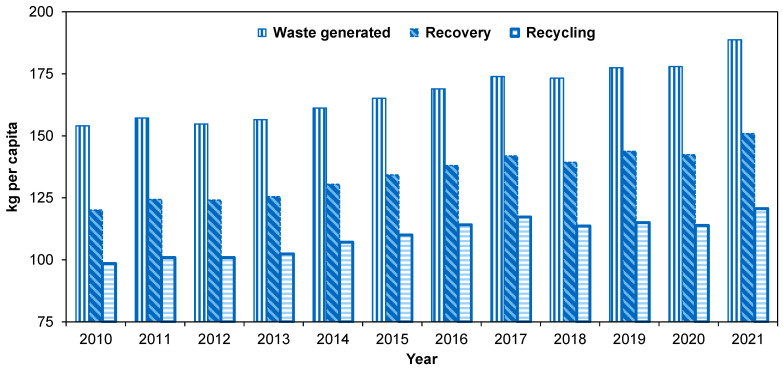
Packaging waste generation, recycling, and recovery in the EU (2010–2021). Values of 2010, 2011, and 2021 are estimates. Source: Eurostat [10].

Concerning energy consumption, using more sustainable energy sources leads to greater energy savings in 3D printing. Furthermore, privileging renewable energy is a more prudent choice for a greener world. With the advancement of technology and science, there is an increase in polymer consumption to meet diverse needs. Table 1 summarizes the utilization of polymers in the European Union (EU) across various sectors. Packing, accounting for 40–45% of the total, followed by construction, with 20–25% of the total, are the two largest consumers. Figure 1 provides a statistical overview of packaging waste generation, recovery, and recycling in the EU for the past 10 years. Total consumption is progressively growing over time, leading to an increase in waste. Addressing this challenge requires additional investment in recycling infrastructures, the development of more efficient recycling technologies, and the implementation of circular economy principles across the plastic packaging lifecycle. As can be seen in the figure, there is a continuous increase in both the generated and recovered waste. Regarding the recycled waste, it experienced an apparent stagnation from 2018 to 2020, followed by an increase in 2021. This shows how crucial recycling is with the growing need over time.

**Table 1:** Utilization of polymers in the EU [3,4,5,6,7,8,9].

Sector	Average Share of Total Polymer Demand (%)	Trends and Observations	Sources
Packaging	40–45	Dominant sector, increased use of biodegradable polymers.	PlasticsEurope, European Bioplastics, Statista.
Construction	20–25	Steady demand, focus on energy-efficient materials.	PlasticsEurope, European Commission Reports.
Automotive	8–10	Lightweighting, growth in engineering plastics.	PlasticsEurope, European Automobile Manufacturers Association.
Electric and electronics	5–7	Growth in consumer electronics, miniaturization.	PlasticsEurope, European Chemical Industry Council (CEFIC).
Agriculture	3–5	Biodegradable films, precision farming technologies.	PlasticsEurope, European Bioplastics.
Consumer goods	10–12	High-performance, aesthetically pleasing products.	PlasticsEurope, Statista, European Bioplastics.

This review underlines the transformative potential of recycled polymers in shaping a more sustainable and responsible future for AM. The focus will mainly be on the integration of recycling polymers to foster a circular economy within the 3D printing industry, contributing to the ongoing efforts towards sustainable practices in additive manufacturing. This paper covers a range of pertinent topics in the field of recycled polymers, such as identifying recyclable and recycled materials; conducting systematic analyses of their properties and characteristics; discussing optimized processes based on case studies; conducting comparative assessments of environmental impacts to enhance sustainability understanding; and providing an overview of relevant regulations, challenges, and future perspectives.

## 2. Recycled Materials for AM

Due to its ease of use and cost-efficiency, additive manufacturing has a huge application area and has received more attention from scientists, researchers, and engineers. Nowadays, filaments, pellets, or granules with recycled polymers are being manufactured and sold by diverse companies for use in AM extrusion processes. Recycled polymers can, thus, have a second life if they are reused in additive manufacturing products [11,12,13]. Current examples include polylactic acid (PLA) [14,15,16], polyethylene terephthalate (PET), acrylonitrile butadiene styrene (ABS), polycarbonate (PC), polypropylene (PP), thermoplastic polyurethane (TPU), thermoplastic elastomer (TPE), high-impact polystyrene (HIPS), polyethylene (PE) [17], polyvinyl chloride (PVC), high-density polyethylene (HDPE) [17], and low-density polyethylene (LDPE) [18], among others. PLA is derived from renewable resources like cornstarch, sugarcane, or tapioca roots, and is a bioplastic known for its biodegradability and recyclability, making it an eco-friendly option. While PLA is a popular choice for 3D printing, recycled PLA is easy to use and can produce versatile lightweight parts. However, as indicated in several studies, despite its good thermal behavior, it generally exhibits lower mechanical properties than virgin PLA [13,19]. Moreover, its source of waste is well known, such as nonused materials or mixed PLA waste, resulting in lower viscosity values, higher crystallization ability, and reduced transparency [20].

For all recycling, including various types of polymers, understanding the provenance of the polymers that will be recycled is crucial. Nevertheless, determining the origin of waste recovered from public containers is not an easy task. Waste from tires and polymers processed by mechanical and cryogenic methods can also be successfully used in 3D printing, even if they show a slightly larger melting point compared to virgin materials [21]. Recycled PET, PE, and PP are mainly originated from plastic bags and packaging, for example, plastic bottles and containers like water bottles, soda bottles, and salad containers. Potential recycling sources can be footwear industries for TPU, tires for TPE, and window frames and thermoformed sheets for HIPS [12]. Commercial recycled ABS polymeric filaments from package food recipients and car dashboards showed no significant differences in mechanical properties compared to their virgin counterparts [19].

In a recent study carried out by Stoof et al. [22], recycled PP from preconsumers was used as base material considering 10%, 20%, and 30% by weight of hemp fiber and harakeke fiber. The results revealed an increase in strength and stiffness for the reinforced PP 3D printing filament reinforced with 30% fiber content. However, the printed parts exhibited significantly reduced values, although they showed superior performance to those of plain PP. PETG is a semitransparent and durable thermoplastic, derived from recycled PET bottles, and it can be reused for 3D printing, offering excellent toughness, chemical resistance, and resistance to warping. Filaments obtained from recycled electronic waste polymers, mostly PC, exhibited a more pronounced degradation in mechanical properties [23].

Nowadays, recycled filaments can be found on the market, created from different sources, including recycled polymer waste, polymer injection molding waste, discarded water bottles, failed 3D-printed parts, etc. In addition, there are also filaments and resins derived from renewable resources, such as corn starch, or recycled materials. These options not only enable materials to be reused but also mitigate the environmental impact of AM processes. Companies such as Dassault Systems (Vélizy-Villacoublay, France) already use eco-friendly filaments [11] and many companies sell recycled filaments to use in 3D printing. Filamentive (Bradford, UK), a UK-based company, offers filaments made from PET and rPET. Reflow Filament (Amsterdam, The Netherlands), a Dutch company, is specialized in recycled filaments derived from plastic waste, including PET, ABS, and PLA. 3D Fuel (Fargo, ND, USA), a USA-based company, provides a wide range of recycled filaments made from coffee, beer, and hemp, as well as polymers PET and ABS. Finally, Filamania Kft (Szigetszentmiklós, Hungary) sells filaments made from PET, ABS, and other materials.

Table 2 shows a list of commercial polymers for AM applications with their respective features and more suitable processing technologies which include fused deposition modeling (FDM), selective laser sintering (SLS), injection, and molding. These polymers can also be recycled to be used in 3D printing. It is interesting to note that there is a vast diversity of polymers available on the market at affordable prices, although the list is not exhaustive. There is a variety of recyclable material sources for 3D printing, reducing the dependence on virgin materials (see Table 2).

Table 3 provides an overview of the available recycled polymers currently available in the market. These materials are second-life polymers, often reinforced with base pure polymer or other materials to improve their properties. Recycling materials not only reduces the cost of the material but also decreases the carbon release and can aid in a greener environment. However, despite its numerous advantages, it is difficult to determine the provenance and composition of the materials from the landfill or waste container, and some polymers cannot be recycled together. More studies have shown that recycled materials face a major problem because their thermal stability changes over multiple recycling, making them useless. To mitigate this problem, materials specifically designed for 3D printing (filaments, pellets, or granules) are being developed by reinforcing and improving their properties with other materials [16,17,18,24,25,26] and optimizing processing parameters [13,27].

The packaging industry heavily relies on recycled PET and HDPE for creating bottles, containers, and packaging films, which helps in reducing waste and resource consumption [31]. The textile industry incorporates recycled PE from discarded articles [32] and PET from staple fiber waste to produce distinct textile parts [32,33]. Recycled polyurethanes can be used in the manufacture of quarter panels, wheel covers, steering wheels, bumper covers, and cores in automotive vehicles [34]. Recycled TPU can be used to print nonpneumatic tires [35]. In building and construction applications, recycled PET can be used to replace aggregates in mixtures as bitumen modifier and mix reinforcement. In addition, recycled PE can be incorporated into asphalt mixtures while recycled polyurethane is utilized as a bitumen improver for waterproofing coatings and as a reactive polymer in polyurethane modified bitumen [36]. Micronized PVC has also been used successfully as modifier for asphalt concrete [36,37].

## 3. Sustainable 3D Printing: Process Optimization and Environmental Impact

**Figure 2:**
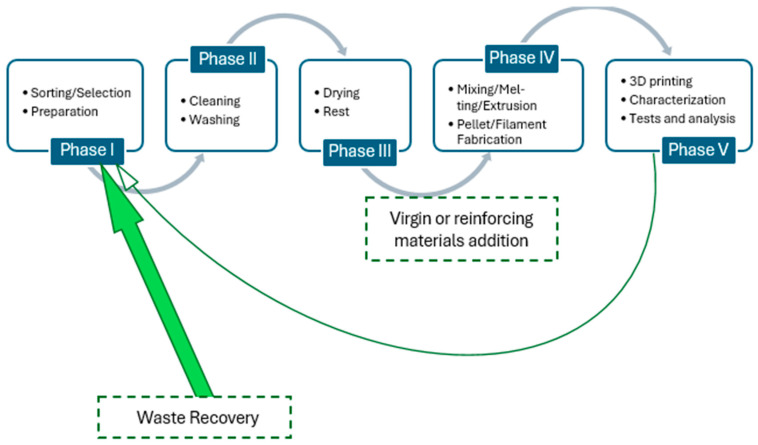
Main steps of the polymer recycling process.

**Figure 3:**
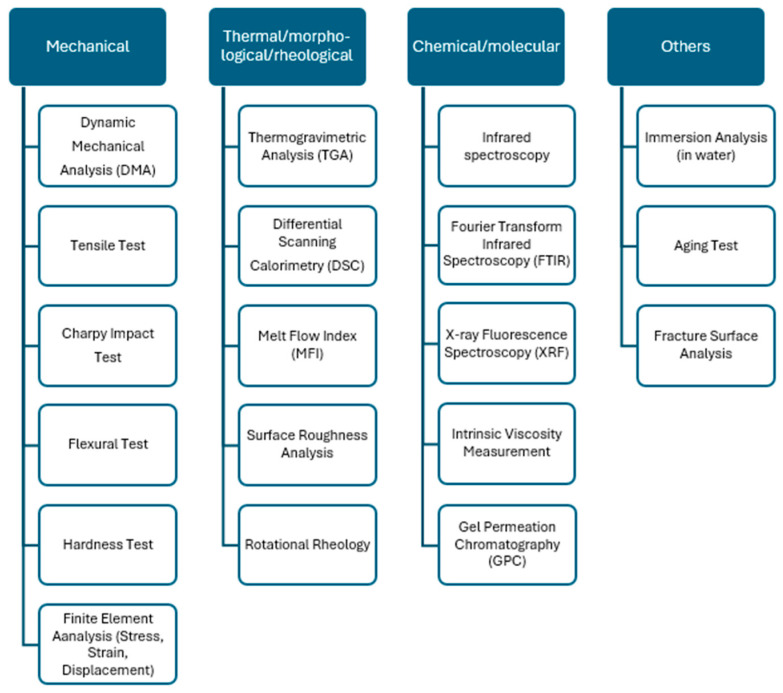
Tests, characterization, and measurements carried out in the recycling process.

Fused filament fabrication (FFF) and FDM processes are the most used 3D printing processes in recycling polymers. Figure 2 summarizes the five-step recycling strategy commonly proposed in the literature. It comprises sorting/selection, cleaning/washing, drying, mixing/melting, extrusion/injection molding, and printing process. The sorting process involves separating different types of polymers from the waste stream. Once sorted, the polymers need to be cleaned to remove contaminants followed by thorough drying to remove any moisture. The drying process may include air drying, hot air drying, or vacuum drying. Next, the mixing/melting process generally involves shredding or granulating polymers into smaller pieces to increase surface area which facilitates melting. During the melting process, virgin or reinforced materials can be added. The molten polymers or composites are then shaped into pellets, filaments, or flake shapes through extrusion/injection molding. Recycled polymer filaments or pellets obtained from the extrusion/injection molding process can be then used as feedstock for 3D printers. The addition of virgin or reinforcing materials is also carried out to improve efficiency. Regarding the methodology used for analyzing the performance of recycling polymers, Figure 3 displays an overview of the most relevant testing procedures and measurement methods found in the literature. For the sake of readability, this information is organized into mechanical testing, thermal/morphological/rheological characterization, chemical/molecular measurement, and other analyses.

Garrido et al. [38] proposed a method for selecting recycled materials to be used in 3D printing, aiming at helping users in materials choice. This selection process is based on multicriteria decision making (MCDM). More specifically, they employed the technique for order preference by similarity to an ideal solution (TOPSIS) which contains six steps: (i) materials are evaluated against criteria depending on properties with the weight of criterion weight set by the user according to the importance of each property; (ii) elements and weights are normalized to ensure they are comparable; (iii) normalized elements are weighted according to the user-defined importance; (iv) according to each criterion which accounts for both benefit and cost, the best and worst materials are identified; (v) the distance of each material to the ideal and nonideal solution is computed using Euclidean distance; and (vi) the closeness of each material to the ideal solution is calculated. This sequential analysis, based on technical criteria, helps users to make more informed decisions by meeting the specific requirements for 3D printing.

Three-dimensional printing is gaining more and more interest in industries, companies, and small organizations. In the fight to reduce pollutants in the sea, Garrido et al. [38] studied the use of 3D-printed recycled materials from maritime plastic waste, including HDPE, LDPE, PET, expanded PS, and PP. They identified two important points in integrating user-oriented 3D printing services: the first is prioritizing decentralized local services, and the second is leveraging external computer-aided manufacturing (CAM) service providers. This solution can also be extended to other fields where there is plastic waste obtained from alternative sources. In another study, Cress et al. [39], through the recycling of ABS, proposed that, in the context of utilizing recycled filament, controllable parameters must be adjusted and continuously measured, for instance, averaging diameter for each batch or filament diameter.

**Figure 4:**
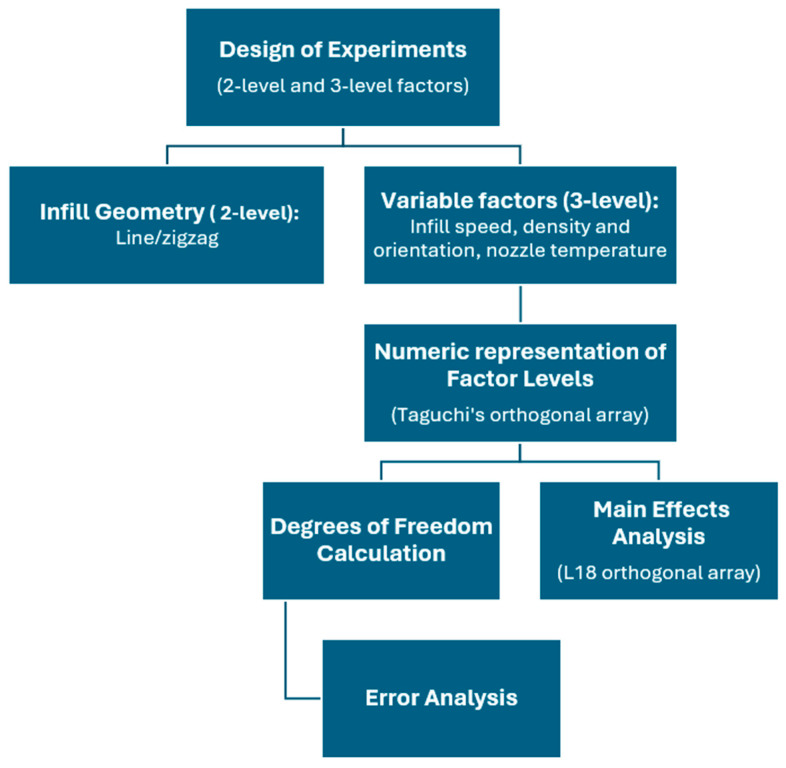
The hierarchical structure of DoE proposed by Gebrehiwot et al. [27] to optimize the mechanical properties of recycled PLA printed by FFF.

**Figure 5:**
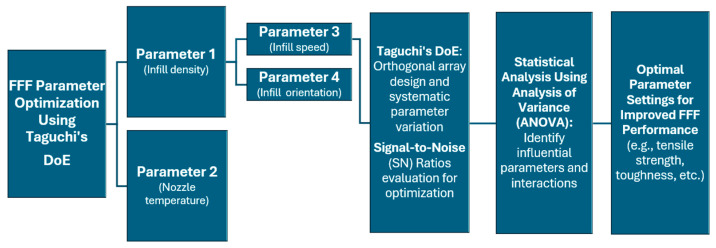
Flow of FFF parameters for the application of Taguchi’s method, systematic parameter variation, statistical analysis, and identification of optimal parameter setting for improved FFF performance [27].

Gebrehiwot et al. [27] studied the effect of infill strategy on the mechanical performance of additively manufactured parts printed through FFF using Reform rPLA supplied by Formfutura. The research aimed to optimize the FFF process parameters by using Taguchi’s method and grey relational analysis. Taguchi’s method, implemented in a single-response approach, indicated that infill orientation and density were the most influential factors in achieving optimum tensile strength while specific combinations of infill geometry, infill density, infill orientation, nozzle temperature, and infill speed influenced the modulus of toughness. Infill orientation and nozzle temperature were highlighted as particularly significant factors affecting the modulus of toughness, while infill density had a lower impact. Grey relational analysis, a multiple-response approach, confirmed the findings of single-response optimization and identified specific parameter levels for achieving optimum responses. Infill orientation was found to be the most influential factor, followed by infill density and nozzle temperature. Based on the above-described optimization procedure, predictive models for tensile strength and modulus of toughness were successfully developed. The study also provided insights into the failure mechanisms associated with the different infill orientations. The practical implications of these findings are significant for optimizing the FFF process as well as achieving the desired mechanical properties in recycled PLA. Figure 4 presents an overview of the design of experiments (DoE), in a step-by-step guide, while Figure 5 shows the application of Taguchi’s DoE proposed in the study carried out by Gebrehiwot et al. [27].

Another research area in the field of sustainable 3D printing processes is the development and optimization of material-recycling machines for reusing polymer materials. Nattukallingal et al. [40] successfully introduced a user-friendly unit, named 3DP-MRM, designed to recycle plastic waste into new filaments, offering an efficient solution for sustainable material utilization in additive manufacturing. Azadani et al. [41] modeled the heat transfer phenomena in an extruder for recycling polymers into filaments for use in additive manufacturing. The main goal of this research was to enhance both the temperature distribution and the cooling rates within the extruder. Wang et al. [35] assessed the performance of 3D-printed nonpneumatic tires and highlighted the importance of optimizing and controlling the printing temperature and the filling percentage for FDM optimization.

Research has explored the reusability of PP, PVC, HDPE, LDPE, PS, PET, ABS, and PC as 3D printing filaments, highlighting the extensive study of PLA due to its natural origin [42].

PLA is the easiest and most recyclable polymer. It is a biomaterial and generally shows a slight decrease in mechanical properties after multiple injection or molding processes compared to virgin PLA [43,44,45]. Furthermore, studies carried out by Agbakoba et al. [46], Gil Muñoz et al. [15], and Tanney et al. [47] provide insights into the quality of recycled PLA. An interesting outcome is that recovered PLA, when reused with the same virgin raw materials, can obtain better thermal stability [15,46,47]. According to Gil Muñoz et al. [15], the recycling process efficiency depends on factors such as the material type, the processing volume, and the presence of contaminants. This conclusion was also reported in the paper by Yoha et al. [48]. After multiple extrusion, ABS and PLA exhibited an increase in melt flow, as observed by Tanney et al. [47]. The assessment performed by Patti et al. [16], based on dynamic mechanical analysis, confirmed minimal impact on thermomechanical properties in 3D-printed parts made of commercially recycled filaments, indicating a slight, but acceptable, level of polymer degradation during the recycling process.

**Figure 6:**
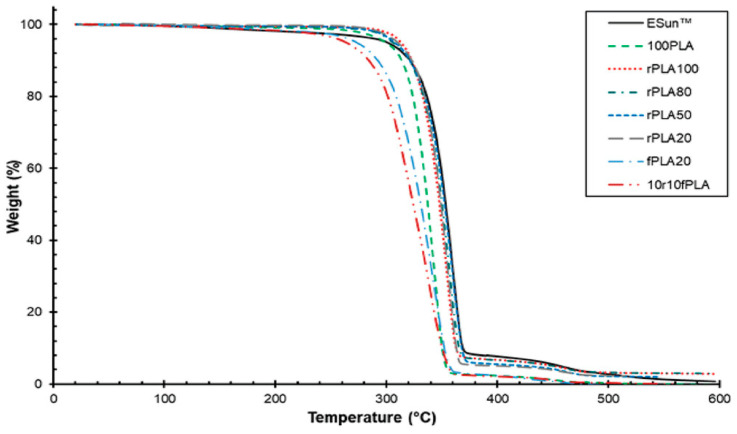
TGA results for different 3D-printed materials. Figure reprinted from reference [46] with permission by Wiley (CC BY).

Agbakoba et al. [46] evaluated the thermomechanical properties of different recycled PLA materials, including those derived from failed 3D-printed components and waste biocomposite filaments. The main features of the tested polymers are summarized in Table 4. The thermogravimetric analysis, displayed in Figure 6, shows that a slight decrease in thermal stability for 100% virgin recycled PLA compared to ESun filament can be observed. In addition, recycled PLA-based filaments with varying blends (e.g., 80% recycled PLA + 20% virgin PLA, 50% recycled PLA + 50% virgin PLA, and 20% recycled PLA + 80% virgin PLA) exhibited improved thermal stability over those without addition (e.g., 100% virgin PLA, and 100% virgin recycled PLA). This confirms the existing literature, suggesting that the addition of pure PLA to recycled variants improves thermal stability.

The DSC results from Agbakoba et al. [46] help in understanding the glass transition behavior, melting point, and crystallinity characteristics of PLA, a semicrystalline material. The 100% virgin PLA has a glass transition temperature that is hardly observed, while the 100% virgin recycled PLA shows a slightly lower glass transition temperature compared to the ESun filament. The other materials (i.e., blended materials, including ESun and 100% virgin PLA, as well as filaments with 20% recycled biocomposite, etc.) show a decrease in glass transition temperature values. The observed decrease in glass transition temperature in recycled PLA indicates potential alterations in molecular structure due to processing or the presence of impurities. Furthermore, the declining trend in glass transition temperature with lower proportions of recycled PLA in blends suggests a potential avenue for fine-tuning material properties by adjusting recycled content levels. Filaments containing recycled materials show an increase in melting temperature compared to ESun filament, indicative of more thermally stable PLA crystallites. In addition, filaments with recycled materials present a higher overall degree of crystallinity, particularly those with nucleating effects from cellulose particles, indicating increased crystallization, such as the 10r10fPLA filament. The addition of virgin PLA to recycled PLA further improved the crystallinity behavior of the materials.

**Figure 7:**
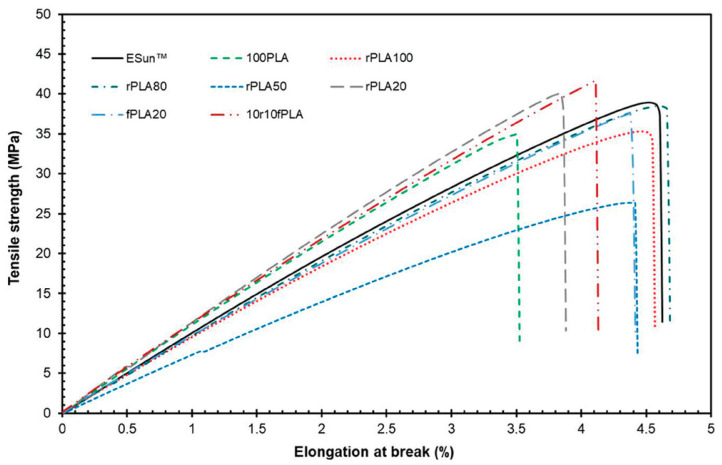
Tensile test results of 3D-printed polymer specimens. Figure reprinted from reference [46] with permission by Wiley (CC BY).

Regarding the monotonic tensile behavior of 3D-printed specimens through FFF (see Figure 7), ESun filaments exhibited the highest ultimate tensile strength (UTS) and maximum force at break (MFb), followed by variations in UTS and MFb for different recycled and virgin PLA filaments. The tensile modulus (TMod) indicated increased stiffness in recycled PLA100 compared to ESun, correlating with higher crystallinity. Moreover, elongation at break (Eb) varied among specimens, with binary blends of recycled and virgin PLA showing improved tensile deformation which was attributed to plasticizers. Fracture behavior analysis indicated brittle fracture for all samples, and DMA revealed a higher initial storage modulus for recycled filament (rPLA100) due to increased crystallinity. Visible permanent deformation was observed for ESun and rPLA100 at the end of the DMA analysis. Agbakoba et al. [46] highlighted the suitability of these recycled PLA materials for 3D printing applications based on their thermal and mechanical properties.

Another successful example of using FFF in recycling PLA is documented in the study by Cruz Sanchez et al. [49]. During five cycles, the polymer (PLA) was printed for four more cycles, even if the mechanical properties of the printed parts decreased due to the 3D printing process. Moreno et al. [20] demonstrated that 3D printing under controlled conditions did not significantly affect the thermal stability of recycled PLA. However, they found that washing and reprocessing steps influenced the intrinsic viscosity of the polymers.

**Figure 8:**
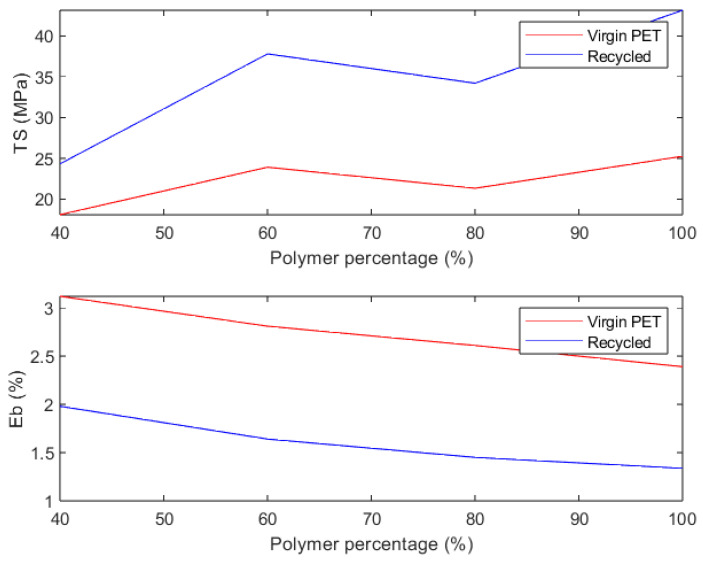
TS and Eb of virgin and recycled 3D printed PET. Data taken from reference [50] with permission by MDPI (CC BY).

Oussai et al. [50] evaluated the mechanical performance of materials made from both recycled PET and virgin PET with four different printing settings (100%, 80%, 60%, and 40% recycled PET) and compared their tensile strength, shear strength, and hardness properties with those from virgin PET. The assessed properties showed minimal variation compared to those of virgin material. There was a 3–9% decrease in the average hardness properties of recycled samples compared to virgin counterparts (see Table 5). However, the result from the shear strength tests of recycled materials showed a 6.8% increase over virgin materials (see Table 5). This unexpected outcome was probably caused by changes at the microscopic level during extrusion and deposition of recycled PET, and the change in Poisson’s ratio led to increased shear strength or the risk for increased emissions of ultrafine particles in recycled PET. The result from the tensile strength tests of recycled materials displayed a 14.7% increase (see Figure 8). The optimal printing setting for recycling PET in 3D printing leading to both the highest tensile strength and elongation at break was found to be 100% recycled PET.

**Figure 9:**
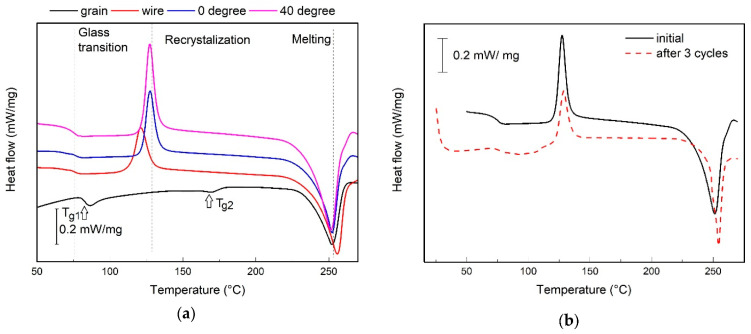
(**a**) Recycled PET grain, filament, and printed specimens at 0° and 40°; (**b**) printed specimen at 40° in an initial state and after three recovery cycles. Figure reprinted from reference [51] with permission by MDPI (CC BY).

Another study by Pricop et al. [51] on recycled PET (see Figure 9) showed a 20% decrease in crystallization degree after three recovery cycles, compared to the initial cycle. In addition, it showed how recycled PET grain, filament, and printed specimens at 0° and 40° can exhibit variations in glass transition, recrystallization, and melting temperature during the first recycling cycle.

Vidakis et al. [52] studied the possibility of using recycled PETG as filament in 3D printing. Continuous recycling of PETG over six cycles showed improved mechanical stability. In the third cycle, the tensile strength was 15.8% higher than pure PETG, and an increase in tensile modulus of elasticity was observed in the fourth cycle. Moreover, PETG maintained its thermal stability up to the degradation onset temperature (~380 °C) across six-cycle rounds, suggesting that the operational temperatures for recycling and 3D printing are safely below this critical temperature to prevent rapid degradation of PETG. Recycling PETG during four-cycle rounds resulted in high-quality printed parts and good layer adhesion with a brittle behavior in the first, fifth, and sixth cycles. Finally, this research highlighted the importance of recycling PETG up to four-cycle rounds to maintain or improve its mechanical properties.

ABS is a tough and versatile thermoplastic extensively used in household products and industrial applications. The use of recycled ABS in 3D printing offers a cost-effective alternative to its virgin counterpart. In a study by Cress et al. [39], the effect of recycling on the polymer chemistry and molecular structure of ABS printed by FDM was assessed via different characterization techniques. This study revealed a substantial overlap in all FTIR peaks, indicating spectral identicality between virgin and recycled ABS within the detection limit of FTIR analysis. A comprehensive characterization through DSC, TGA, FTIR, and GPC revealed no discernible alteration in the molecular structure of recycled ABS, even after multiple recycling cycles. However, XRF analysis revealed a progressive increase in iron content with each recycling iteration. The tensile strength decreased during the third cycle, which may be caused by the presence of increased variation of porosity in the recycled ABS. The study conducted by Pinho et al. [19] reported that recycled black ABS offers a thermal stability similar to the virgin material with a slight difference in glass transition temperature. These authors also reported that recycled ABS exhibits smoother surfaces and higher value of contact angle. Concerning their mechanical properties, a slight increase was observed in both the tensile strength and the modulus of elasticity as well as a lower elongation strain at break which was associated with the introduction of additives.

Pan et al. [17] presented the effect of additives on properties of recycled PP/HDPE. Recycled PP/HDPE with the addition of nanocrystalline powders of iron (Fe), silicon (Si), and aluminum (Al) showed a more uniform morphology with fewer cracks compared to the one with Fe–Si and Fe–Si–chromium (Cr) addition. Furthermore, recycled PP/HDPE with Fe–Si–Al and Fe–Si–Cr showed an improvement in thermal stability. The analysis of its mechanical properties showed that recycled PP/HDPE with Fe–Si–Al has the best yield strength, and recycled PP/HDPE with Fe–Si–Cr/Al has a good Young’s modulus. The addition of metal powders resulted in shortening the plastic regime of the recycled materials. However, the elastic regime increased, resulting in a stronger filament.

Garrido et al. [38] addressed the possibility of using recycled polymers from waste in marine as a second life in 3D printing. They demonstrated that the addition of additives can increase the flexibility of PP and reduce its overall shrinkage coefficient which can contribute to improved printability of recycled PP. Domingues et al. [21] studied recyclable PP blended with tire wastes. The thermal properties of the blended materials assessed through STA showed a crystallization temperature of virgin PP lower than the recycled PP–tires blended material and a slightly closer melting temperature. However, the recycled PP–tires blended material presented two melting peaks; the first was related to the melting of one constituent of the tires and the second was related to the polymer fusion.

Vidakis et al. [53] assessed the mechanical response of PP over multiple recycling processes (six cycles). The results showed a reduction in the ultimate tensile strength, and an increase of 45% in both the flexural strength and modulus of elasticity. The second-cycle sample showed the highest thermal stability, while the six-cycle sample showed the lowest thermal stability. The two samples after the third and sixth cycles showed a single melting peak, while the sample after the second cycle exhibited an additional small peak around 130 °C, indicating the presence of β-polypropylene crystals.

Singh et al. [25] investigated the utilization of recycled HDPE and Nylon 6 for bearing application. It was found that different proportions of zirconium oxide (ZrO_2_) reinforcement can significantly affect the flow properties and microstructure of recycled HDPE and Nylon 6. The MFI values for Nylon 6 varied with the different proportions of ZrO_2_, generally decreasing with increased proportion of ZrO_2_. In addition, increased ZrO_2_ content also made Nylon 6 composites more difficult to melt and flow. The MFI values of HDPE with additions up to 40% ZrO_2_ were moderate, keeping these materials suitable for FDM. However, higher ZrO_2_ proportions further reduce the MFI values, indicating less melt flowability. The corresponding microstructures showed the formation of fine grains for HDPE-ZrO_2_ with the finest grains for the Nylon 6-ZrO_2_ matrix. The HDPE-40% ZrO_2_ combination gave the lowest coefficient of friction, emphasizing its resistance to wear environments and its suitability for bearing applications. In another study, Singh et al. [18] investigated recycling LDPE as base material and SiC/Al_2_O_3_ as reinforcements. The MFI values of LDPE-SiC/Al_2_O_3_ were slightly higher as the reinforcement percentage increased (30%, 20%, and 10%). An interesting outcome was that 100% LDPE gave the lowest MFI value.

Wang et al. [35] investigated the performance of 3D-printed nonpneumatic tires made from recycled TPU. They highlighted the necessity to print this type of polymer before its decomposition temperature. The tests performed to evaluate its wear resistance and fatigue strength showed that recycled TPU material is a very good choice. It was also noticed that when the 3D filling percentage increases, the printed parts exhibit higher tensile strength, at the specific temperature of 210 °C, with fewer voids. In another study carried out by Plummer et al. [54], during repeated recycling of TPU, the tensile strength showed a slight decrease and a small overall increase in melt flow rate corresponding to a decrease in viscosity. The decrease in viscosity is an interesting outcome for SLS application. However, it can decrease the mechanical properties of recycled TPU. Nanni et al. [55] evaluated the mechanical behavior of recycled TPU over time, up to 50 days. They observed that there was no significant decrease in mechanical properties even if the material is subjected to degradation phenomena. The study confirmed the possibility of recycling TPU multiple times. Recycled TPU experienced diverse simulated environmental conditions, such as exposure to ultraviolet rays, heat, and humidity. The result showed that recycled TPU can resist up to a certain period without significant loss of properties. In the same context, Vidakis et al. [56], through multiple recycling of TPU, showed that there were no significant differences in thermal properties after six cycles. On the other hand, the results of tensile tests showed an increase in both tensile strength and modulus of elasticity in the sixth cycle, contrary to the impact tests which led to decreasing values as the number of cycles increased. From the fractured surface analysis, a brittle behavior was observed up to the fourth cycle. However, the last two cycles exhibited a ductile behavior.

The use of recycled PC in 3D printing was evaluated by Gaikwad et al. [23]. These authors assessed the mechanical behavior of recycled PC made from e-waste filament and 3D-printed parts. E-waste filament exhibited a ductile behavior at both lower stress and lower elongation, and a brittle fracture just after necking. The SEM images showed rougher fracture surfaces. Recycled PC exhibited a tensile strength equal to 83% of the virgin PC but with higher flexibility. However, it showed lower resistance to deformation. Recycled PC showed a higher thermal conductivity which resulted in rapid cooling during printing and in reduced mechanical properties. They also reported a decrease in thermal stability over recycling. Recycling resulted in a reduction in the mechanical performance of PC, but despite this, recycled PC is also a viable solution.

Romani et al. [57] tested both recycled PC and blended recycled PC/ABS (30% weight of rABS). The tested parts showed a similar mechanical behavior and the failure was characterized by brittle mechanisms. The increased impact energy and increased mechanical resistance of rPC/ABS were attributed to the presence of rABS. Comparatively, the fractured surface of rPC was considerably homogeneous without visible voids. Reich et al. [58] evaluated the mechanical performance of recycled PC printed using different 3D printing systems: Gigabot X with pellet extrusion/melt extrusion (PME/FGF), FFF-based Gigabot, and FFF-based TAZ. The tensile testing results indicated that the recycled TPU material, when printed using the different 3D printing systems, exhibited mechanical properties comparable to those of commercial filaments. Compression testing results revealed significant differences in the performance of samples printed with the Gigabot X and FFF-based TAZ printers. Samples printed using the FFF-based TAZ printer showed higher compressive stresses compared to those printed using the Gigabot X printer.

Three-dimensional printing with recycled materials contributes to sustainability by reusing materials, instead of relying solely on virgin resources. To comprehensively evaluate the environmental impact, a life cycle assessment is recommended, with a focus on recyclability [59]. This should consider factors such as raw material extraction, manufacturing, transportation, product use, and end-of-life management [53]. A study by Oladapo et al. [60] highlighted the fact that recycling in 3D printing consumes less energy and generally leads to lower greenhouse gas emissions because of the energy requirements depending on the type of material, recycling process, and energy source. Schwarz et al., 2021 [59] advocated that the environmental impact of recycling depends on treatment methods, with a trade-off between treatment intensity and the positive impact of avoided products; and highlighted that improving recycled product quality and quantity is crucial for reducing overall environmental impact. Even if recycling 3D printing does not consume more energy, the energy sources used and the recycling processes have an impact on the environment. Oladapo et al. [60] added that achieving net-zero carbon emission depends on factors such as energy consumption, economic viability, and materials to be recycled. Another study by Kreiger et al. [61] highlighted the impact of transportation on energy consumption related to the recycling of HDPE affecting the environment. Conventional recycling was found to be less environmentally friendly when compared with distributed recycling, and the kg CO_2_ equivalent per kg HDPE showed a substantial reduction of 89% in greenhouse gas emissions for distributed recycling compared to virgin material. Using recycled PET over PLA has been found to contribute to a more sustainable and eco-friendly 3D printing process [62].

## 4. Considerations on Regulation and Standards

Regulation and standards for the sustainability of manufacturing processes is an important challenge that requires concerted efforts from policymakers, industry stakeholders, and the public to promote innovation, invest in infrastructure, and adopt robust regulatory measures. Feeley et al. [63] worked on the development of an ethical product standard for 3D printing filaments. Their approach involved integrating established fair-trade standards, life cycle analysis of the embodied energy and emissions associated with recycled filament production and 3D printing manufacturing, and extensive consultations with partners and stakeholders. The accreditation standards for “ethical filament” follow a similar approach to other fair-trade standards. They include baseline requirements for accreditation while incorporating additional requirements that evolve with industry advancement. These standards encompass distinct criteria, such as minimum pricing, application of fair-trade premiums, labor regulation, environmental considerations, and health and safety protocols, as well as social standards covering discrimination, harassment, freedom of association, collective bargaining, and discipline practices.

**Figure 10:**
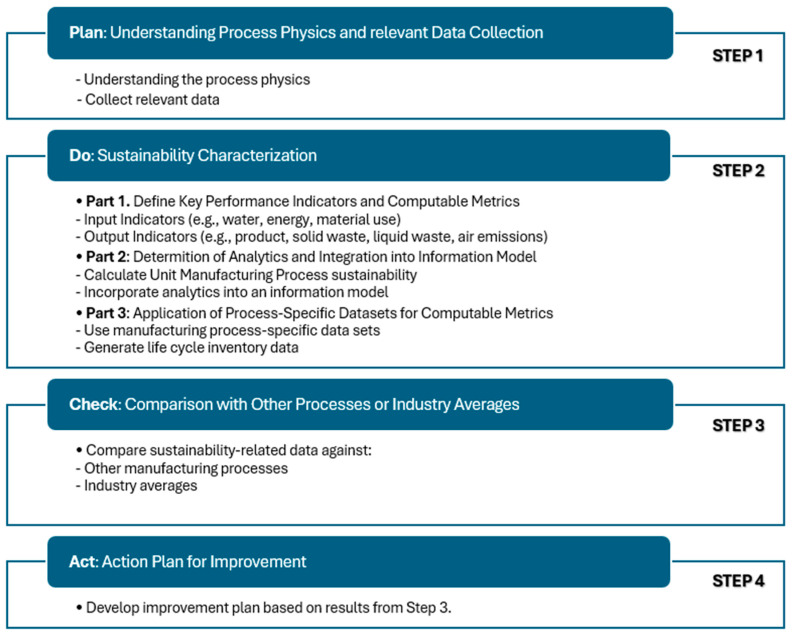
Characterization methodology for a sustainable process within the ASTM E60.13 standards committee [64].

Mani et al. [64] proposed the sustainability characterization methodology summarized in Figure 10. The main steps, as displayed in the figure, comprise plan, do, check, and act. The planning step involves understanding the physics of the AM process (e.g., energy sources, melting and solidification, etc.) and collecting relevant information accordingly. In the second step, defining the key performance indicators and the analytics before applying the process-specific datasets is primordial. The next step encompasses checking the data obtained from the sustainable characterization and comparing them with other processes. The last step includes the action plan for improvement. The authors presented compiled ASTM standards related to AM. Additive manufacturing technology standards established by ASTM Committee F42 [65] play a crucial role in advancing the AM industry. These standards are instrumental in promoting knowledge dissemination, stimulating research efforts, and facilitating technology implementation within the AM sector. The standards address several topics, including terminology, production process performance, end-product quality, and machine calibration. They are continuously refined by technical subcommittees within ASTM Committee F42, and the National Institute of Standards and Technology (NIST) in the USA [66,67]. Moreover, the collaborative efforts between the ASTM F42 Committee and the ISO Technical Committee 261 hold new prospects for advancing recycling initiatives in AM, favoring a future more marked by sustainability [67,68]. An interesting discussion on the lack of standards governing quality assurance and quality control in AM can be found in the paper by Kietzmann et al. [69].

Rejeski et al. [67] highlighted the need for research in waste management and life cycle assessment. To meet industry requirements, it is essential to implement robust standardization and quality control measures. Regulations and standards play an important role in protecting product safety, ensuring high quality, and guaranteeing compliance with environmental regulations [69]. Currently, regulations and standards related to the use of recycled materials in 3D printing are limited. Existing regulations such as Registration, Evaluation, Authorization, and Restriction of Chemicals (REACH) [66,70] primarily focus on ensuring that hazardous substances present in base materials are properly managed and minimized when used in recycled materials. In addition, the ASTM F3091/F3019M-14 standard for Powder Bed Fusion of Plastic Materials also addresses these concerns [68].

ASTM and ISO [71] are actively developing standards for the use of recycled materials in 3D printing. In the same way, the American Chemical Society [72] is focused on establishing standards that detail the characterization of recycled materials, including their chemical composition and physical properties. Additionally, the Society of Plastics Engineers (SPE) [73] is working on standards for the design and testing of 3D-printed structures created from recycled materials.

## 5. Challenges and Future Prospects

### 5.1. Challenges Identification in Using Recycled Polymers

The use of recycled polymers in 3D printing represents a set of several challenges such as variability in material properties, quality control, traceability, and market limitations. They have a large range of properties due to the diverse types of polymers and other kinds of materials present in the recycling process, making it difficult to predict the final properties of the printed parts. The lack of standardized testing methods [67] for recycled polymers hinders ensuring the required quality of the materials used in 3D printing. Furthermore, recycled polymers often originate from complex supply chains with limited traceability, which makes it difficult to track their provenance and chemical composition. In addition, the current market for 3D-printed products derived from recycled polymers remains restricted due to insufficient awareness regarding the benefits of recycled materials and concerns about product quality and performance.

Despite recycling being a good solution to give a new life to material waste, multiple recycling cycles can cause fluctuation in material properties. The possible deterioration in the mechanical properties of PLA after repeated recycling may limit the usability of the material, potentially resulting in increased waste if the degraded material cannot be effectively recycled [74]. However, some recent studies have explored solutions to this issue by reinforcing the polymer with virgin material or by adding other reinforcing materials [17,18,24,26,27,47,75]. The variation of properties has been confirmed through mechanical, thermal, and morphological analyses of recycled parts.

The abovementioned changes depend on various factors, including crystallinity, polymer chain shortening, and molecular weight distribution, among others. Vidakis et al. [36] emphasized the importance of considering these factors when evaluating the suitability of recycled PP for specific applications. They have also worked on the suitability and mechanical response of recycled PETG and PLA [52,74]. Further investigations, including X-ray diffraction (XRD), are recommended to better understand alterations in crystalline structure [53]. Other studies have highlighted the challenges associated with achieving optimal printability for recycled PP and PET [22,50], due to issues such as bed adhesion, deformation, and weak interfacial welding between printed layers. The success of the printing process is closely linked to the cooling process and the diffusion of polymer chains between layers, with common failures occurring at these interfaces. Additionally, there has been limited progress in recycling conventional thermosets for AM [76]. Tian et al. [24] emphasized that the price of recycled material exceeds that of virgin material and that the additive manufacturing process of a part generally consumes more time compared to traditional methods, such as casting, extrusion, fabrication, or injection molding. Furthermore, the formation of voids is commonly listed as a significant defect that results in inferior and anisotropic mechanical properties of parts produced through FDM, the most used process in recycling polymers [77,78].

### 5.2. Potential Advancements and Future Directions

The world is trying to adopt greener practices to reduce carbon emissions and other pollutants. In this perspective, efforts are being made to explore more sustainable solutions. An alternative approach to address this issue is to consider the utilization of biodegradable materials since the 3D printing process is a promising tool in this regard due to its inherent advantages. However, we cannot just focus on the advantages; AM also has challenges. Thus, potential advancements are needed for future directions. On this matter, Oladapo et al. [60] elaborated a list of recommendations for the sustainability of filament recycling and for achieving net-zero emissions such as the (i) adoption of renewable energy sources in the recycling process to minimize greenhouse gas emissions; (ii) implementation of efficient transportation systems and optimizing logistics to reduce transportation-related emissions; (iii) development of policy incentives and promotion of consumer awareness to encourage the use of recycled filament; and (iv) investment in research and development to improve the recycling process and the quality of recycled filaments.

Oliveira et al. [79] recommended further research focused on optimizing the printing orientation for improved results and to mitigate its impact on mechanical performance. Scanlon et al. [80] concluded that mixing and melting for recycling polymers before the extrusion process could improve issues related to low intrinsic viscosity. Furthermore, to address challenges and accelerate the adoption of recycled polymers in 3D printing, it is essential to consider better traceability of recycled polymers throughout the supply chain and further develop new applications for 3D-printed products made from recycled polymers.

## 6. Conclusions

This review highlighted studies exploring the application of recycled polymers in AM and their role in achieving net-zero carbon emissions and promoting economy circular methodology implementation. The integration of recycled polymers into AM processes holds promise for advancing sustainability in the manufacturing sector. Despite the challenges associated with this technology, namely, material variability as well as regulatory limitations, ongoing research and development efforts have been identifying the potential benefits of utilizing recycled materials in 3D printing. Collaborative initiatives aimed at enhancing material traceability, establishing ethical standards, and optimizing recycling processes are crucial for maximizing the environmental and economic benefits of recycled polymers in AM. Moving forward, continued investment in research, policy incentives, and industry collaboration will be fundamental to unlocking the full potential of recycled polymers in achieving a more sustainable and environmentally friendly manufacturing ecosystem.

## Figures and Tables

**Table 2:** Commercially available recyclable materials for specific use in 3D printing.

Material	Type of Material	Printing Methodologies	Price per kg (EUR)	Company	Features
PET	Pellets/Flakes	FDM, injection, molding	1.70–2.20	Carbon LITE (Los Angeles, CA, USA), Loop Industries (Terrebonne, QC, Canada), Envision Plastics (Reidsville, NC, USA	High transparency, lightweight, and strong.
HDPE	Pellets	FDM, injection, molding	1.50–1.80	KW polymers (Troy, AL, USA), green Line polymers (Waterloo, IA, USA), MBA Polymers (Hackensack, NJ, USA)	High strength, stiffness, and chemical resistance.
PP	Pellets	FDM, injection, molding	1.20–1.80	LyondellBasell (Rotterdam, The Netherlands)	Lightweight, high chemical resistance, and low moisture absorption.
ABS	Pellets	FDM, injection, molding	1.50–2.00	Total Petrochemicals (Houston, TX, USA), INEOS Styrolution (Frankfurt, Germany)	High-impact resistance, strength, and toughness.
PLA	Pellets	FDM	1.60–1.90	Nature Works (Minneapolis, MN, USA), 3D FUEL (Fargo, ND, USA)	Biodegradable, derived from renewable resources.
Nylon	Pellets	FDM, SLS	1.80–2.10	BASF (Ludwigshafen, Germany), Evonik (Essen, Germany)	High strength, flexibility, and durability.
PEEK	Pellets	FDM, SLS	1.60–2.00	Solvay (Brussels, Belgium)	High-temperature resistance, chemical resistance, and stiffness.
TPU	Pellets	FDM	2.50–3.50	Lubrizol (Wickliffe, OH, USA)	Flexible, durable, and abrasion-resistant.
PC	Pellets	FDM, injection, molding	1.80–2.20	Covestro (Leverkusen, Germany), SABIC (Riyadh, Saudi Arabia)	High impact resistance, optical clarity, and heat resistance.
PS	Pellets	FDM, injection, molding	2.00–2.50	INEOS Styrolution (Frankfurt, Germany), BASF (Ludwigshafen, Germany)	Lightweight and rigid.

**Table 3:** Recycled polymers available on the market. PLA: polylactic acid, PETG: polyethylene terephthalate glycol modified, PPGF: thermoplastic reinforced with glass fiber.

Material	Type of Material	Origin	Price (EUR)	Company	Advantages	Source
PLA	Filaments	Production waste	26.50	EUMAKERS (Barletta, Italy)	Not specified.	[28]
PLA	Filaments in different colors	Factory waste streams	20–22	Filamentive (Bradford, UK)	Low warping, limited smell, and good print quality.	[29]
PLA	Filaments in different colors	Food packaging waste	26.32	Reflow Filament (Amsterdam, The Netherlands)	Easy to print, durable, and excellent print quality.	[30]
rPETG	Filaments	Leading local recyclers	28	Reflow Filament (Amsterdam, The Netherlands)	Durable and easy to use, exceptional visual, and mechanical performance.	[30]
rPPGF	Filaments	Fishing nets and ropes reinforced with glass fiber	Not specified	Reflow Filament (Amsterdam, The Netherlands)	Anti-warping, and excellent UV and chemical resistance.	[30]

**Table 4:** Recycled PLA from different sources. Table reprinted from reference [46] with permission by Wiley (CC BY).

S/N	Filament	Description	Filament Diameter [mm]
1	ESun	Commercial filament	1.72 (±0.03)
2	100PLA	100% virgin PLA	1.65 (±0.02)
3	rPLA100	100% virgin recycled PLA	1.67 (±0.11)
4	rPLA80	80% recycled PLA + 20% virgin PLA	1.65 (±0.12)
5	rPLA50	50% recycled PLA + 50% PLA	1.67 (±0.10)
6	rPLA20	20% recycled PLA + 80% virgin PLA	1.64 (±0.08)
7	fPLA20	20% recycled biocomposite filament f + 80% virgin PLA	1.70 (±0.02)
8	10r10fPLA	10% rPLA + 10% fPLA, +80% virgin PLA	1.68 (±0.07)

**Table 5:** Shear strength and hardness of virgin and recycled 3D printed PET [50].

Polymer Type	Shear Strength (MPa)	Hardness (Shore D)
Virgin	28.45 ± 0.69	73.10 ± 0.73
Recycled	29.25 ± 2.00	68.71 ± 2.00

## Data Availability

The raw data supporting the conclusions of this article will be made available by the authors on request.

## Nomenclature

3D	Three-dimensional
ABS	Acrylonitrile butadiene styrene
AM	Additive manufacturing
ASTM	American Society for Testing and Materials
CAM	Computer-aided manufacturing
CNC	Computer numerical control
DMA	Dynamic mechanical analysis
DoE	Design of experiments
DSC	Differential scanning calorimetry
Eb	Elongation at break
EU	European Union
FDM	Fused deposition modeling
FEA	Finite element analysis
FFF	Fused filament fabrication
FTIR	Fourier-transform infrared spectroscopy
GPC	Gel permeation chromatography
HDPE	High-density polyethylene
HIPS	High-impact polystyrene
ISO	International Organization for Standardization
LDPE	Low-density polyethylene
MT	Metric ton
MFb	Maximum force at break
MFI	Melt flow index
NIST	National Institute of Standards and Technology
PC	Polycarbonate
PE	Polyethylene
PEEK	Polyether ether ketone
PET	Polyethylene terephthalate
PETG	Polyethylene terephthalate glycol modified
PLA	Polylactic acid
PP	Polypropylene
PPGF	Thermoplastic reinforced with glass fiber
PS	Polystyrene
PVC	Polyvinyl chloride
r	Material containing recycled polymer
REACH	Registration, evaluation, authorization, and restriction of chemicals
SEM	Scanning electron microscope
SLS	Selective laser sintering
SPE	Society of Plastics Engineers
TGA	Thermogravimetric analysis
TMod	Tensile modulus
TPE	Thermoplastic elastomer
TPU	Thermoplastic polyurethane
TS	Tensile strength
USA	United States of America
UTS	Ultimate tensile strength
XRD	X-ray diffraction
XRF	X-ray fluorescence
